# **Welfare Chauvinism, Populist Radical Right Parties and Health Inequalities** Comment on "A Scoping Review of Populist Radical Right Parties’ Influence on Welfare Policy and its Implications for Population Health in Europe"

**DOI:** 10.34172/ijhpm.2020.149

**Published:** 2020-08-10

**Authors:** Clare Bambra, Julia Lynch

**Affiliations:** ^1^Population Health Sciences Institute, Faculty of Medical Sciences, Newcastle University, Newcastle Upon Tyne, UK.; ^2^Department of Political Science, University of Pennsylvania, Philadelphia, PA, USA.

**Keywords:** Politics, COVID-19, Social Policy, Public Health

## Abstract

In this short commentary, we examine the implications of the welfare chauvinism of the populist radical right (PRR) for health inequalities by examining the international evidence about the impact of previous periods of welfare state contraction on population health and health inequalities. We argue that parties from various political traditions have in fact long engaged in stigmatisation of welfare recipients to justify welfare state retrenchment, a technique that the PRR have now ‘weaponised.’ We conclude by reflecting on implications of the rise of the PRR for the future of welfare states and health inequalities in the context of coronavirus disease 2019 (COVID-19).

In their scoping review, Rinaldi and Bekker^1^ examine the welfare policy consequences of the rise of populist radical right (PRR) parties in Europe and the implications for population health. They conclude that the exclusionary welfare chauvinistic positions of PRR parties are likely to have negative effects on access to welfare and healthcare provision, adversely impacting the health of vulnerable population groups. Whilst their review is wide-ranging and covers various PRR policy mechanisms, one of the key factors that Rinaldi and Bekker identify is the PRR division of welfare recipients into ‘deserving’ and ‘undeserving’ – with knock-on impacts on the overall size, shape and acceptability of public welfare provision for everyone. In this short commentary, we examine the implications of this in more detail by examining the international evidence about the impact of previous periods of welfare state contraction on population health and health inequalities. We argue that parties from various political traditions have in fact long engaged in stigmatisation of welfare recipients to justify welfare state retrenchment, a technique that the PRR have now ‘weaponised.’ We conclude by reflecting on implications of the rise of the PRR for the future of welfare states and health inequalities in the context of coronavirus disease 2019 (COVID-19).

PRR parties – including the Rassemblement National (French National Front), the Austrian Freedom Party, the Italian Northern League, the Alternative for Germany, the Polish Law and Justice Party, the Dutch Party for Freedom, the UK Independence Party, the Finns party and the Sweden Democrats – are nationalist/nativist, authoritarian and populist (privileging the ‘common sense’ of ‘the people’ over elite knowledge).^2^ Their approach to the welfare state has been described as ‘welfare chauvinism’ because it involves increasing or defending welfare provisions (notably social security and healthcare) for the native-insider population whilst limiting access and eligibility for outsider groups – most notably immigrants and ethnic, religious, cultural, and linguistic minorities (although the wider PRR agenda also includes reducing the rights of LGBTQ+ [lesbian, gay, bisexual, transgender, queer, and others] minorities and women’s reproductive rights).^2^ Welfare chauvinism links native birth or ethnicity (and sometimes other attributes related to religion, culture, and language) to moral ‘deservingness,’ which entitles those who possess it – and only those – to state support in time of need.^3^ So, PRRs in government *can* lead to an increase in welfare state generosity.^4^ However, deservingness criteria can also be used to restrict welfare provision for other individuals and groups in the population, too, and are seen for example in policies that aim to reduce welfare ‘dependency’ amongst those characterized as shiftless, improvident, or otherwise undeserving of social support.^1^

The implications of the linkage of nativity with deservingness for the health of minority groups is as straightforward as it is awful. Minority ethnic groups have worse health than the native population – for example, they have higher rates of hypertension, diabetes, asthma, heart, liver, renal disease, cancer, cardiovascular disease, obesity and smoking.^5^ And yet, as Rinaldi and Bekker point out in their review, the influence of PRR welfare chauvinism has led to calls for – and in some countries such as the UK implementation of – restrictions on access to healthcare and welfare state support for immigrant communities.^1^ This has huge public health implications – not just for the health of the excluded population groups, but also, in the context of endemic infectious disease (notably tuberculosis and COVID-19), for the entire population. The nativist and authoritarian combination within PRR welfare chauvinism also results in restrictions on welfare state access and social citizenship for lower socio-economic groups within the native/insider community themselves. The trope of deserving and undeserving recipients is used to further retrench the welfare state for everyone – particularly in relation to unemployment support and pension provision.^1^

The tactic of splitting welfare recipients into deserving and undeserving is not novel or exclusively one of the PRR.^6^ Initially based in Protestant charity doctrine, and hence less prevalent in Catholic countries,^7^ there is nevertheless a long history in social policy in Europe and other high income countries of distinguishing between deserving insiders (eg, hard-working families, widows) and undeserving outsiders (eg, scroungers, shirkers, unwed mothers). This is most notable in the liberal Anglosphere,^8^ but elements of deservingness narratives are also visible in the social policies of the Nordic countries, for example.^9^ Such narratives have been cultivated and put to use for decades - largely by the mainstream political right (eg, Conservative, Republican, Liberal, and Christian Democrat parties) but also in some cases by Social Democratic parties – to justify cutting the welfare state *for everyone*.^8,9^ Notable examples of this are in the United Kingdom where unemployment, lone parent and even disability benefits were ‘reformed’ (retrenched) by successive governments of both the mainstream political right and left from the 1980s onwards.^10^ Benefit values relative to wages (replacement rates) in the United Kingdom fell (eg, the replacement value of unemployment benefit decreased from 45% of average wages in 1980 to just 16% in 2000), entitlement restrictions and increased qualifying conditions reduced coverage (the population coverage of unemployment benefit in the United Kingdom decreased from 90% in 1980 to 77% in 2000), duration of benefit receipt were considerably shortened, and most recently, sanctions were introduced for those who fail to meet increasingly strict entitlement criteria.^11^ These changes – amounting to a recommodification of labour – were reflected to a greater or lesser extent in other countries.^11^ For example, in Germany the replacement value of unemployment benefit decreased from 68% of average wages in 1980 to 37% in 2000, and in Norway from 70% to 62%.^11^ Similarly, in the United States (where deservingness is also highly racialised^12^) reductions in welfare support and increasing work requirements targeted at ‘undeserving’ minority lone parents (‘welfare queens’) were found to have negative health impacts on mothers and their children.^13^ More broadly, both hostility toward non-natives and reductions in social protections in the United States – beginning in the 1980s and continuing through the post-global financial crisis (GFC) recession – have resulted in increasing health inequalities and worsening population health, including rising ‘deaths of despair’ among white Americans^14^ (conversely, expansions of generosity and eligibility in specific programs had beneficial effects on health^15,16^). The PRR have merely newly ‘weaponised’ what are unfortunately long-standing and divisive political and cultural narratives.

The impact of these substantial reductions in the generosity and universality of welfare state programs on health inequalities has been empirically examined in multiple countries.^17^ For example, studies have found that health inequalities increased when significant cuts to the welfare state and public spending were implemented by many European countries as a response to the 2008 GFC. For example, in England, studies have found that inequalities in mental health and well-being increased at a higher rate between 2009 and 2013, with people living in more deprived areas experiencing the largest increases in poor mental health and self-harm.^18^ Similarly, increases in child poverty since the implementation of austerity in England were associated with increased inequalities in infant mortality rates (IMRs) (deaths aged under 1 year), with every 1 per cent increase in child poverty associated with an extra 5.8 infant deaths per 100000.^19^ Inequalities in IMR, life expectancy, and mortality amenable to healthcare in England also increased from 2010 onwards.^20,21^ Across Europe, reductions in spending levels and increased conditionality may have adversely impacted the mental health of disadvantaged social groups.^22^

These findings about the effects of austerity on health inequalities are in keeping with previous studies of the effects of public sector and welfare state contractions in the United Kingdom, the United States, and New Zealand in the 1980s and 1990s. Krieger et al found that inequalities in premature mortality (deaths under age 75) and IMRs by income and ethnicity increased in the United States between 1980 and 2002 – a period when Republican right-wing governments (starting with Ronald Reagan 1980-1988) cut public welfare services (including healthcare insurance coverage) and reduced social assistance benefit levels.^23^ Similarly, research into the health effects of Thatcherism in the United Kingdom (1979-1990 – right-wing Conservative government) found that the welfare state retrenchment pursued in this period was accompanied by increased socio-economic inequalities in life expectancy and IMR.^24^ These findings are mirrored in studies of welfare state reductions in New Zealand which found that while general mortality rates declined, socioeconomic inequalities amongst men, women, and children in all-cause mortality increased in the 1980s and the 1990s during a period in which New Zealand underwent major structural changes (including more targeted social benefits, privatisation of public housing, user charges for welfare services).^25^ Even in the later-liberalizing countries of continental Europe, the dominance since the 1990s of a neoliberal master-narrative that privileges budgetary restraint and limited state action has hampered efforts to reduce health inequalities.^26^ Population health as a whole has suffered, too: Welfare provision is not just beneficial for the health of the most disadvantaged and marginalised, but also for the whole population, reducing total mortality and increasing life expectancy. ^11,17^

This body of work provides the best insights into the potential impact of PRR welfare chauvinism on the health of vulnerable and lower socio-economic groups. It makes for grim reading: welfare chauvinism is yet another lever for scaling back the welfare state, resulting in increasing health inequalities. The COVID-19 pandemic may well further enhance the political influence of the PRR. Nativist discourses and authoritarian measures (eg, the first post-war lockdown across Europe including closed borders) have become increasingly mainstreamed during the pandemic, and are likely to worsen as the economic ramifications of the pandemic, including mass unemployment expected across Europe and other high-income countries, take hold. A widespread economic depression is likely to increase health inequalities, especially if further welfare state retrenchment is enacted as a result.^27^ The PRR may well contribute to – and benefit from - economic volatility by further promoting welfare chauvinism and protectionist trade policies. This will test the legal and constitutional barriers (eg, in the European Union where the European Court of Justice is a strong defender of cross-border welfare rights) that currently offer some protection against welfare chauvinist policies from being fully enacted. Beyond welfare chauvinism, other notable areas of public health policy that have been beneficial for reducing health inequalities are under threat from PRR parties, include tobacco control (eg, the Austrian coalition government incorporating the PRR Austrian Freedom Party cancelled the planned public smoking ban) and reproductive health rights (eg, in the United States, Trump is championing the restriction of access to abortions and birth control).^28,29^ As the Rinaldi and Bekker review shows, it is increasingly pressing for public health and health policy researchers and policy makers to understand the potential threats posed by the PRR and welfare chauvinism for increasing health inequalities.^1^

## Ethical issues

 Not applicable.

## Competing interests

 Author declares that he has no competing interests.

## Authors’ contributions

 CB led on conception and design, analysis and interpretation, drafting of the manuscript and critical revision of the manuscript. JL contributed to analysis and interpretation, drafting of the manuscript and critical revision of the manuscript.

## Authors’ affiliations

^1^Population Health Sciences Institute, Faculty of Medical Sciences, Newcastle University, Newcastle Upon Tyne, UK. ^2^Department of Political Science, University of Pennsylvania, Philadelphia, PA, USA.
